# Different murine-derived feeder cells alter the definitive endoderm differentiation of human induced pluripotent stem cells

**DOI:** 10.1371/journal.pone.0201239

**Published:** 2018-07-26

**Authors:** Masaki Shoji, Hiroki Minato, Soichiro Ogaki, Masahide Seki, Yutaka Suzuki, Shoen Kume, Takashi Kuzuhara

**Affiliations:** 1 Laboratory of Biochemistry, Faculty of Pharmaceutical Sciences, Tokushima Bunri University, Tokushima, Japan; 2 School of Life Science and Technology, Tokyo Institute of Technology, Kanagawa, Japan; 3 Department of Computational Biology and Medical Sciences, Graduate School of Frontier Sciences, University of Tokyo, Chiba, Japan; University of Kansas Medical Center, UNITED STATES

## Abstract

The crosstalk between cells is important for differentiation of cells. Murine-derived feeder cells, SNL76/7 feeder cells (SNLs) or mouse primary embryonic fibroblast feeder cells (MEFs) are widely used for culturing undifferentiated human induced pluripotent stem cells (hiPSCs). It is still unclear whether different culture conditions affect the induction efficiency of definitive endoderm (DE) differentiation from hiPSCs. Here we show that the efficiency of DE differentiation from hiPSCs cultured on MEFs was higher than that of hiPSCs cultured on SNLs. The qPCR, immunofluorescent and flow cytometry analyses revealed that the expression levels of mRNA and/or proteins of the DE marker genes, SOX17, FOXA2 and CXCR4, in DE cells differentiated from hiPSCs cultured on MEFs were significantly higher than those cultured on SNLs. Comprehensive RNA sequencing and molecular network analyses showed the alteration of the gene expression and the signal transduction of hiPSCs cultured on SNLs and MEFs. Interestingly, the expression of non-coding h*XIST exon 4* was up-regulated in hiPSCs cultured on MEFs, in comparison to that in hiPSCs cultured on SNLs. By qPCR analysis, the mRNA expression of undifferentiated stem cell markers *KLF4*, *KLF5*, *OCT3/4*, *SOX2*, *NANOG*, *UTF1*, and *GRB7* were lower, while that of *hXIST exon 4*, *LEFTY1*, and *LEFTY2* was higher in hiPSCs cultured on MEFs than in those cultured on SNLs. Taken together, our finding indicated that differences in murine-feeder cells used for maintenance of the undifferentiated state alter the expression of pluripotency-related genes in hiPSCs by the signaling pathways and affect DE differentiation from hiPSCs, suggesting that the feeder cells can potentiate hiPSCs for DE differentiation.

## Introduction

Human embryonic stem cells (hESCs), and human induced pluripotent stem cells (hiPSCs) can differentiate into varous types of cells found in human organs, such as the brain, liver, heart, pancreas, lung, and the small intestine [1–7]. As hESCs are associated with several ethical issues, hiPSCs are now expected to be a valuable tool for predicting the clinical safety and efficacy of drug candidates, or for clinical application of regenerative medicine. Undifferentiated hiPSCs can be induced to differentiate into the three principal germ cell layers, ectoderm, mesoderm, and definitive endoderm (DE), by different methods, thereby forming the various cells of human organs [1, 4, 7]. Thus, to obtain a large number of organ-specific differentiated cells from hiPSCs, it is important to maintain the proper undifferentiated state of the hiPSCs and to induce their efficient differentiation into the three principal germ layers.

The growth of undifferentiated hiPSCs is typically maintained by culturing the cells on a murine-derived feeder cell layer and with stem cell medium supplemented with basic fibroblast growth factor (bFGF) in a conventional culture method [1, 3, 4, 7]. The murine-derived feeder cell layer usually comprises SNL76/7 feeder cells (SNLs) [1, 3], which are mouse fibroblast STO cells transformed with murine leukemia inhibitory factor (LIF) and neomycin resistance genes [8], or mouse primary embryonic fibroblast feeder cells (MEFs) [2, 7, 9]. Both are mitotically inactivated by treatment with mitomycin C or γ-ray irradiation prior to use. Tomoda *et al*. [10] reported that female hiPSCs cultured on SNLs have two active X chromosomes (XaXa), whereas female hiPSCs cultured on MEFs have an Xa and one inactive X chromosome (Xi) (XaXi). The authors also showed that early passage hiPSCs have XaXi and that XaXi hiPSCs can be converted to XaXa hiPSCs upon more than 15 passages on SNLs. In addition, Ojala *et al*. [4] cultured a single female hESC line (H7) and three male hiPSC lines (UTA.00112.hFF, UTA.00106.hFF, and UTA.00525.LQT2) under three different culture conditions: SNLs and MEFs combined with conventional stem cell medium and on the matrigel matrix combined with mTesR1 medium i.e feeder-free culture conditions [11]. They further compared the mesoderm-derived cardiac differentiation efficiency and found that culturing on SNLs and MEFs promoted cardiac differentiation of hESCs and hiPSCs and inhibited ectoderm-derived neuronal differentiation when compared with feeder-free culture conditions. Interestingly, the female ESC line H7 and male iPSC line UTA.00525.LQT2 showed higher efficiency of cardiac differentiation when cultured on MEFs than when cultured on SNLs, indicating differences in the efficiency of mesoderm differentiation of hESCs and hiPSCs depending on the type of feeder cell employed. Thus, differences in culture conditions between SNLs and MEFs affect the active/inactive status of the X chromosome in female hiPSCs and the efficiency of mesoderm differentiation. However, the efficiency of DE differentiation of hiPSCs is still unclear.

In this study, we cultured the most widely used female hiPSC lines 201B7 and 253G1 [3, 12] on SNL and MEF to evaluate their undifferentiated state and efficiency of differentiation into DE, depending on the type of feeder cell used. We found that the mRNA and protein expressions of the DE marker genes of the sex-determining region Y-box 17 (SOX17) and Forkhead box A2 (FOXA2) and the expressions of the DE surface marker C-X-C chemokine receptor type 4 (CXCR4) [7, 13, 14] were lower in the DE cells induced from 201B7 and 253G1 cells cultured on SNLs than in those induced from 201B7 and 253G1 cells cultured on MEFs under culture conditions employed for DE differentiation. These results suggested that undifferentiated culture of hiPSCs on SNLs inhibited DE differentiation when compared with MEFs. The comprehensive RNA sequencing and molecular network analyses showed that the feeder cells affect the genes expressions of hiPSCs. In addition, exon 4 of human X inactive specific transcript (h*XIST*), which plays a major role in X chromosome inactivation [15, 16], was up-regulated in 201B7 cells cultured on MEFs, in compared with that on SNLs. Compared with 201B7 cells cultured on MEFs, the mRNA expression levels of Krüppel-like factors (*KLF4* and *KLF5*), octamer-binding transcription factor (*OC*T) *3/4*, sex-determining region Y-box 2 (*SOX2*), nanog homeobox (*NANOG*), undifferentiated embryonic cell transcription factor 1 (*UTF1*), and growth factor receptor-bound protein 7 (*GRB7*) genes of 201B7 cells cultured on SNLs were increased, whereas left-right determination factor 1 (*LEFTY1*) and *LEFTY2* genes mRNA expression were decreased. Altogether, we show that differences in the culture conditions of SNLs or MEFs for maintenance of the undifferentiated state alter the expression of pluripotency-related genes by the defined-signaling pathways and X chromosome inactive status, which then affects DE differentiation from hiPSCs.

## Materials and methods

### Culture of human induced pluripotent stem cells

**Fig 1:**
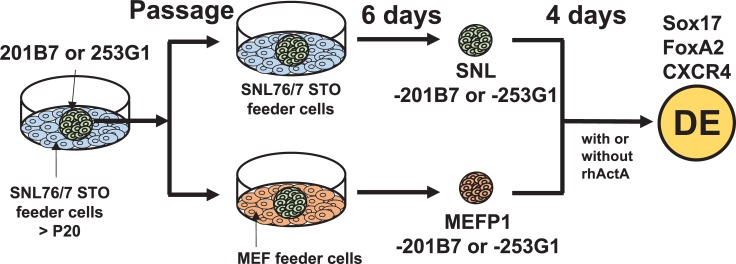
Experimental procedure for DE differentiation from SNL- and MEFP1-201B7 and -253G1 cells. hiPSC line 201B7 or 253G1 colonies that had been passaged more than 20 times on SNLs (>P20) were passaged on SNLs (SNL-201B7 and -253G1) and MEFs (MEFP1-201B7 and -253G1) and then cultured for 6 days in basic fibroblast growth factor (bFGF)-supplemented stem cell medium. SNL- and MEFP1-201B7 and -253G1 cells were seeded in Matrigel-coated wells. The medium was changed to DE differentiation medium supplemented with 1% dimethyl sulfoxide (DMSO) and 100 ng/ml recombinant human activin A (rhActA) and the cells were incubated for 4 days. Medium without rhActA was used as a negative control.

**Fig 2:**
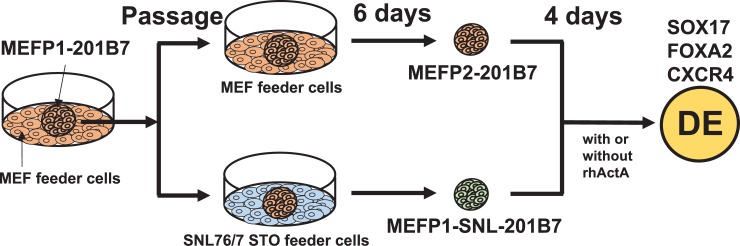
Experimental procedure for DE differentiation from MEFP2- or MEFP1-SNL-201B7 cells. The MEFP1-201B7 colonies in Fig 1 were passaged on MEFs (MEFP2-201B7) or SNLs (MEFP1-SNL-201B7) and then cultured for 6 days. The MEFP2- and MEFP1-SNL-201B7 cells were seeded in Matrigel-coated wells. The medium was changed to differentiation medium supplemented with 1% DMSO and 100 ng/ml rhActA and the cells were incubated for 4 days. Medium without rhActA was used as a negative control.

The hiPSC lines 201B7 [3] and 253G1 [12] (Cell No. HPS0063 and HPS0063) were purchased from the Riken Bioresource Center cell bank (Ibaraki, Japan). Undifferentiated hiPSCs were maintained on mitomycin C (Kyowa Hakko Kirin, Tokyo, Japan)-treated SNLs (DS Pharma Biomedical, Osaka, Japan) for more than 20 passages (>P20) at 37°C and 5% CO_2_. 201B7 and 253G1 colonies cultured on SNLs for a prolonged time were passaged on SNLs (SNL-201B7 and -253G1) or on mitomycin C-treated MEFs (MEFP1-201B7 and -253G1) and cultured for 6 days at 37°C and 5% CO_2_ (Fig 1). In addition, MEFP1-201B7 cells were passaged on mitomycin C-treated MEFs (MEFP2-201B7) and SNLs (MEFP1-SNL-201B7) and cultured for 6 days at 37°C and 5% CO_2_ (Fig 2). The hiPSC were passaged after the removal of feeder cells using CTK solution [2.5% trypsin (Life Technologies, CA), 1 mg/ml collagenase IV (Life Technologies), 0.1 M CaCl_2_, and 20% knockout serum replacement (Life Technologies) in H_2_O]. All undifferentiated hiPSCs were grown in primate embryonic stem cell medium or ReproStem medium (ReproCELL, Kanagawa, Japan) supplemented with 4 ng/ml recombinant human bFGF (rhbFGF, Wako, Osaka, Japan) and 50 U/ml penicillin with 50 μg/ml streptomycin (P/S, Life Technologies).

### Definitive endoderm differentiation

DE differentiation from hiPSCs was induced as per a previously reported method [7]. Briefly, 201B7 and 253G1 colonies cultured on SNLs and MEFs (Figs 1 and 2) were treated with 10 μM Y-27632, an inhibitor of Rho-associated coiled-coil forming kinase (Wako, Osaka, Japan), for 24 hr at 37°C and 5% CO_2_. SNLs or MEFs were removed from these colonies cultured plates using CTL solution. Then these colonies were washed with PBS(-) and dissociated into single cells using 0.05% trypsin-EDTA (Life Technologies, CA). Subsequently, 5 × 10^4^ cells per well were seeded in a 96-well plate coated with BD Matrigel matrix (BD Bioscience, NJ) and cultured in 4500 mg/l glucose Dulbecco’s modified Eagle medium (DMEM) supplemented with 10% fetal bovine serum (FBS, Hyclone Laboratories, IA), 100 μM MEM nonessential amino acid solution (NEAA, Life Technologies), 2 mM l-glutamine, 100 μM 2-mercaptoethanol (2-ME, Life Technologies), and P/S (Life Technologies). After incubation for 24 hr at 37°C and 5% CO_2_, the cells were washed with phosphate-buffered saline (PBS), and the medium was changed to differentiation medium [4500 mg/l glucose DMEM supplemented with 1% dimethyl sulfoxide (DMSO) Hybri-max (Sigma, St. Louis, MO), 2% B-27 supplement vitamin A (Life Technologies), 100 μM NEAA (Life Technologies), 2 mM l-glutamine, 100 μM 2-ME (Life Technologies), and P/S (Life Technologies)] with or without 100 ng/ml recombinant human activin A (rhActA) (R&D Systems, MN, USA) and the cells were incubated for 4 days at 37°C and 5% CO_2_. The medium was replaced every 2 days with fresh medium.

### Quantitative real-time PCR (qPCR)

Total RNA was extracted from cell lysates using an RNeasy Mini Kit (Qiagen GmbH, Germany). Total RNA was used to synthesize cDNA using SuperScript VILO (Life Technologies) according to the manufacturer’s instructions. The synthesized cDNA was used as a template for qPCR, which was performed using a SYBR Green real-time PCR Master Mix (TOYOBO, Osaka, Japan). The primers used for the undifferentiated cells, DE marker genes and *hXIST* RNA are shown in S1 Table [3, 7, 14, 17, 18]. PCR and data analyses were performed on an Applied Biosystems StepOne Plus real-time PCR system (Life Technologies). Relative expression was calculated by the ΔΔCT method. The expression levels of each mRNA were normalized to those of the human housekeeping gene glyceraldehyde 3-phosphate dehydrogenase (*GAPDH*).

### Immunofluorescent staining

Cells were fixed with 4% paraformaldehyde in PBS for 30 min at 4°C prior to permeabilization with 0.3% Triton X-100 for 20 min at 25°C. The cells were incubated with primary antibodies against proteins of undifferentiated stem cell markers or the DE marker gene products SOX17 and FOXA2 (described in S2 Table) [3, 7, 17] at 4°C overnight and then with secondary antibodies (described in S3 Table). Cell nuclei were stained using diamidino-2-phenylindole (DAPI, Life Technologies). Wells were photomicrographed using a fluorescence microscope (*BIOREVO* BZ-9000 or BZ-X700, Keyence, Osaka, Japan). The percentage of SOX17 or FOXA2-positive cells per DAPI-positive cells was calculated based on the numbers of DE marker-positive cells and DAPI-positive cell numbers measured with BZ-II or X analyzers (Keyence).

### Flow cytometry

Cells were dissociated into single cells using 0.05% trypsin-EDTA (Life Technologies). The cells were suspended in Stain Buffer (FBS) (BD Pharmingen, NJ) and then stained with the DE surface marker phycoerythrin (PE)-conjugated mouse anti-human CD184 (CXCR4) or an IgG2a antibody (12G5, BioLegend, CA) [7] for 2 hr at 4°C. A PE-conjugated mouse IgG2a antibody (MOPC-173, BioLegend) was used as an isotype control. Dead cells were excluded using 7-amino-actinomycin D (7-AAD) (BioLegend). The stained cells were analyzed using a Guava easyCyte flow cytometer (Millipore, Darmstadt, Germany), and the data were analyzed using FlowJo software (Tree Star, OR).

### Enzyme-linked Immunosorbent Assay (ELISA)

The concentrations of mouse LIF and ActA protein in the culture medium of SNLs or MEFs were examined by mouse LIF and ActA ELISA. Briefly, 2.5 × 10^5^ mitomycin C-treated SNLs or MEFs were seeded in a 0.1% gelatin (Sigma)-coated 60-mm dish in 4500 mg/l glucose DMEM supplemented with 7% FBS and P/S (Life Technologies) at 37°C and 5% CO_2_. After a 24-hr incubation, the medium was changed to 4 ml of ReproStem medium (ReproCELL) supplemented with 4 ng/ml rhbFGF (Wako) and P/S (Life Technologies). The supernatant of each culture medium was collected and centrifuged and replaced with fresh medium every 24 hr for 6 days. The concentrations of mouse LIF and ActA protein in the collected culture medium were measured using mouse LIF and human/mouse/rat ActA Quantikine ELISA kits (R&D Systems) according to the manufacturer’s instructions.

### Alkaline Phosphatase Staining (ALP)

The cells were fixed with 4% paraformaldehyde in PBS for 10 min at 25°C. After being washed with water, ALP staining was performed using a Leukocyte Alkaline Phosphatase Kit (Sigma) according to the manufacturer’s instructions [3].

### Reverse Transcription PCR (RT-PCR)

For RT-PCR, 5 × 10^4^ cells were seeded in Matrigel matrix (BD Bioscience, NJ)-coated 96-well plates after the removal of feeder cells and incubated for 24 hr at 37°C and 5% CO_2_, following which, total RNA was extracted using an RNeasy Mini Kit (Qiagen). Total RNA was used to synthesize cDNA using SuperScript IV (Life Technologies) according to the manufacturer’s instructions. The synthesized cDNA was used as a template for PCR, which was performed using AmpliTaq Gold DNA Polymerase (Life Technologies); the undifferentiated stem cell marker gene-specific primers used are shown in S3 Table [3, 17]. Human housekeeping gene *GAPDH*-specific primer and no-RT controls were used as positive and negative controls, respectively. PCR products were analyzed by agarose electrophoresis and stained with ethidium bromide.

### Transcriptome analysis by comprehensive RNA sequencing using next generation sequencing

We used RNA sequencing (RNA Seq) to conduct a comprehensive transcriptome analysis in SNL- and MEFP1-201B7 cells, using the method previously reported by Kanematsu *et al*. [19]. Briefly, 5 × 10^4^ cells (n = 6) were seeded in Matrigel-coated 96-well plates after the removal of feeder cells, and incubated for 24 hr at 37°C and 5% CO_2_ following which total RNA was extracted using an RNeasy Mini Kit (Qiagen). The mRNA-sequencing libraries were constructed from each total RNA extract using the SureSelect strand-specific RNA library preparation kit (Agilent Technologies), according to the manufacturer’s instructions. Thirty-six-base pairs of single end reads were generated using an Illumina Hiseq3000 sequencer (Illumina). To exclude reads originating from rRNA, RNA-Seq reads aligned to rRNA by bowtie2 were removed [20]. Using Tophat2, the remaining reads were mapped to the human reference genome sequence (UCSC hg38) [21]. The reads that were uniquely mapped to the genome and whose mapping score was not less than fifty were utilized in the following analysis. The reads per kilobase per million mapped reads (RPKM) were calculated from the number of reads mapped to the exons of RefSeq transcripts. The complete RNA sequencing transcriptome analysis has been deposited in the DNA Data Bank of Japan database (accession number DRA006179). Ratios of each gene in Refseq database were calculated using the RPKM averages in SNL- and MEFP1-201B7 cells.

### Molecular network and pathway analysis

Differentially expressed genes were identified by statistical evaluation and p-values were calculated (S4 Table). Histogram statistics were generated for the dataset of comprehensive RNA sequences (see the bottom of S4 Table). We excluded genes whose normalized values (RPKM value/RPKM of *GAPDH*) were less than 0.0005, because they include considerable noise. Subsequently, we identified genes whose expression ratios (MEFP1-201B7 cells / SNL-201B7 cells) were greater than 1.5 or less than 2/3. We judged that genes with a *p* value of < 0.01 were considered significant. As a result, we found that 222 genes satisfied these criteria. The molecular network of these differentially expressed 222 genes was analyzed using a data-mining tool named KeyMolnet originally developed by KM Data Inc., Tokyo, Japan [22]. KeyMolnet constitutes a knowledge-based content database of numerous interactions among genes, molecules, diseases, pathways, and drugs. The “common upstream” search enabled us to extract the most relevant molecular network composed of those genes coordinately regulated by putative “common upstream” transcription factors. The extracted molecular network was compared side-by-side with distinct canonical pathways of the KeyMolnet library, which includes a broad range of signal transduction pathways, metabolic pathways, and transcriptional regulations. The statistical significance in concordance between the extracted network and the canonical pathways was evaluated by an algorithm that counts the number of overlapping molecular relations shared by both. This makes it possible to identify the canonical pathway exhibiting the most significant contribution to the extracted network. The calculation of significance score is based on the following formula: O = the number of overlapping molecular relations between the extracted network and the canonical pathway, V = the number of molecular relations located in the extracted network, C = the number of molecular relations located in the canonical pathway, T = the number of total molecular relations installed in KeyMolnet, and X = the sigma variable that defines coincidence.

$$\mathrm{Score}=-\log_{2}\left(Score\;(p)\right)$$

$$\mathrm{Score}\;(p)=\sum_{X=0}^{Min(C,V)}f(x)$$

$$f(x)={}_{c}C_{X}\times {}_{T-C}C_{V-X}÷{}_{T}C_{V}$$

### Statistical analysis

All results are expressed as the mean ± standard error of the mean (SEM). The statistical significance of differences between two groups was analyzed by Student’s t test, whereas that between more than two groups was analyzed by one-way analysis of variance (ANOVA). Results were considered significantly different at p < 0.05.

This research was approved by Tokushima Bunri University Review Board.

## Results

### Differences in DE differentiation of hiPSCs cultured on SNL and MEF feeder cells

To evaluate the efficiency of DE differentiation of hiPSCs cultured on SNLs and MEFs, we prepared the female hiPSC lines 201B7 and 253G1 on these different feeder cell layers, SNL-201B7 and -253G1 or MEFP1-201B7 and -253G1 (Fig 1). We further induced DE differentiation of SNL- and MEFP1-201B7 and -253G1 cells using dimethyl sulfoxide (DMSO) and recombinant human activin A (rhActA) supplemented medium (Fig 1) [7].

**Fig 3:**
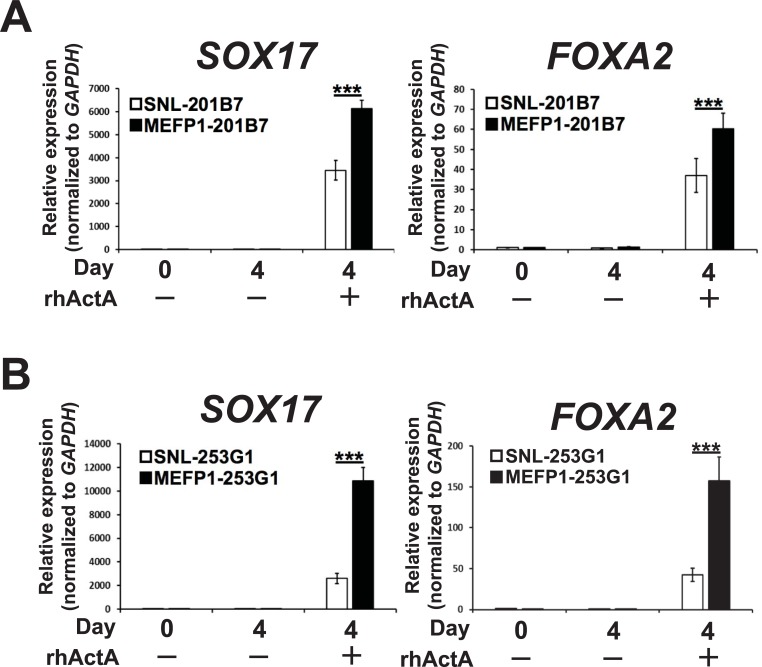
Expression analysis of *SOX17* and *FOXA2* mRNA in DE differentiated SNL-201B7 and -253G1 or MEFP1-201B7 and -253G1 cells by RT-qPCR. The relative mRNA expression levels of *SOX17* (201B7, n = 9 each and 253G1, n = 8 each) (A and B, left panels) and *FOXA2* (201B7, n = 18 each or 253G1, n = 8 each) (A and B, right panels) were determined by RT-qPCR, normalized to those of *GAPDH*, and expressed in relation to levels of DE differentiated SNL-201B7 and -253G1 cells observed at day 0 (set as 1). Data represent the mean ± SEM and are representative of three experiments. ***p < 0.001 versus SNL-201B7 and -253G1 cells.

**Fig 4:**
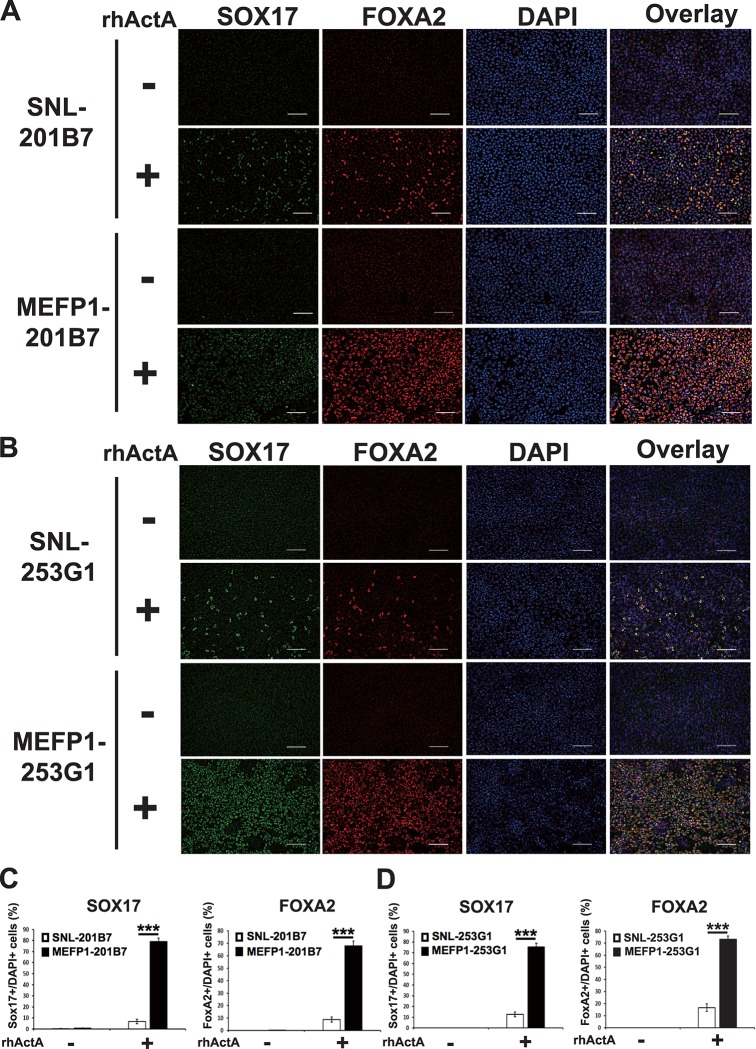
Immunofluorescent staining of SOX17 and FOXA2 proteins in DE differentiated SNL-201B7 and -253G1 or MEFP1-201B7 and -253G1 cells. The images of SOX17 (green) and FOXA2 (red), and DAPI (blue) are shown (A and B). White scale bars, 100 μm. The percentages of SOX17 (C and D, left panels) and FOXA2 (C and D, right panels)-positive cells per DAPI-positive cells (201B7, n = 10 each and 253G1, n = 8 each) were calculated based on DE marker-positive and DAPI-positive cell numbers. Data represent the mean ± SEM and are representative of three experiments. ***p < 0.001 versus SNL-201B7 and -253G1 cells.

**Fig 5:**
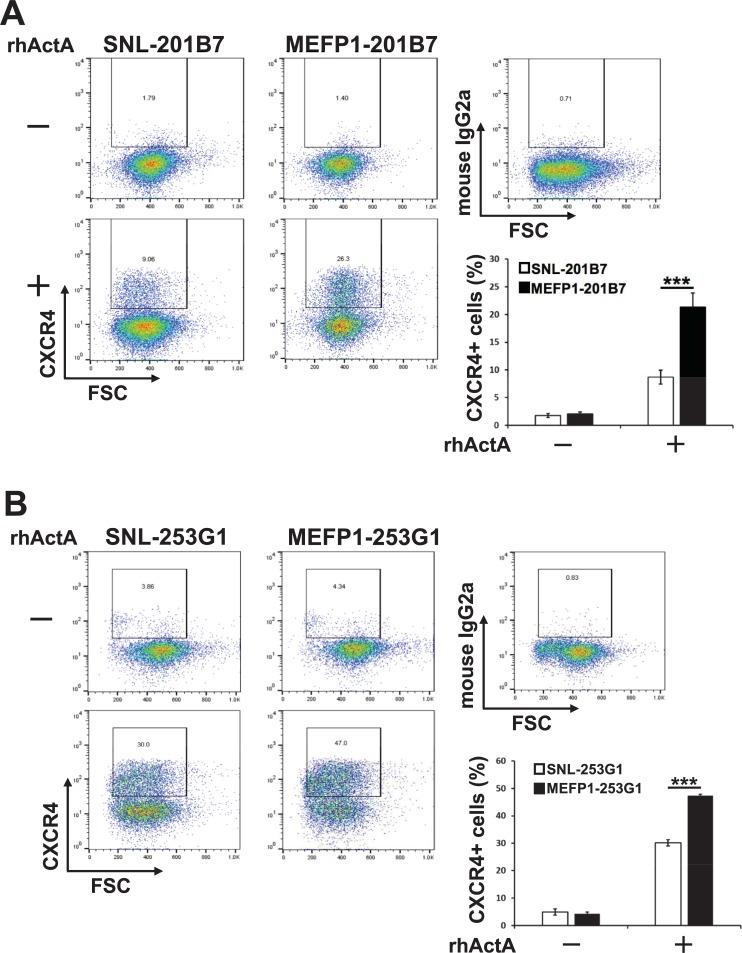
Flow cytometric analysis of CXCR4 protein expression in DE differentiated SNL-201B7 and -253G1 or MEFP1-201B7 and -253G1 cells. The percentages of CXCR4-positive cells in DE differentiated SNL-201B7 (A) and -253G1 (B) or MEFP1-201B7 (A) and -253G1 cells (B) (n = 12 each) were analyzed by flow cytometry. Mouse IgG2a antibody was used as an isotype control. Dead cells were excluded by using 7-AAD. Data represent the mean ± SEM and are representative of more than two or three experiments. ***p < 0.001 versus SNL-201B7 and -253G1 cells.

After a 4-day incubation, the efficiency of DE differentiation was examined by analyzing the expression levels of *SOX17* and *FOXA2* mRNAs (Fig 3) and proteins (Fig 4), and of the CXCR4 protein (Fig 5). In the presence of rhActA, the mRNA expression levels of *SOX17* (Fig 3A and 3B, left panel) and *FOXA2* (Fig 3A and 3B, right panel) were significantly lower in the DE cells differentiated from SNL-201B7 or -253G1 cells than in the DE cells differentiated from MEFP1-201B7 or 253G1 cells. In contrast, in the absence of rhActA, no differences were observed in the expression levels of *SOX17* and *FOXA2* mRNAs between the SNL- and MEFP1-201B7 or -253G1 differentiated cells (days 0 and 4) (Fig 3A and 3B) before and after the medium change. The immunofluorescent expressions of SOX17 and FOXA2 in the DE cells differentiated from SNL- and MEFP1-201B7 or -253G1 cells with and without rhActA were photomicrographed and analyzed (Fig 4). There were a low number of SOX17- and FOXA2-expressing DE cells differentiated from SNL-201B7 and -253G1 cells (Fig 4A and 4B) and the percentages of SOX17- and FOXA2-positive cells per total cells (marked with DAPI, cell nuclei staining) were significantly lower for DE cells differentiated from SNL-201B7 and -253G1 cells (Fig 4C and 4D) than for MEFP1-201B7 and -253G1 cells in the presence of rhActA. In addition, the percentage of CXCR4-positive DE cells differentiated from SNL-201B7 and -253G1 cells was significantly lower than that of CXCR4-positive DE cells differentiated from MEFP1-201B7 and -253G1 cells (Fig 5).

**Fig 6:**
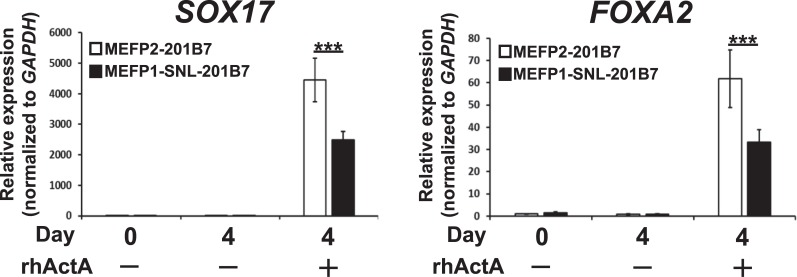
Expression analysis of *SOX17* and *FOXA2* mRNAs in DE differentiated MEFP2- and MEFP1-SNL-201B7 cells by RT-qPCR. The relative mRNA expression levels of *SOX17* (n = 9 each) (left panel) and *FOXA2* (n = 9 each) (right panel) were determined by RT-qPCR, normalized to those of *GAPDH*, and expressed in relation to levels of DE differentiated day 0 MEFP2-201B7 cells (set as 1). Data represent the mean ± SEM and are representative of three experiments. ***p < 0.001 versus MEFP2-201B7 cells.

**Fig 7:**
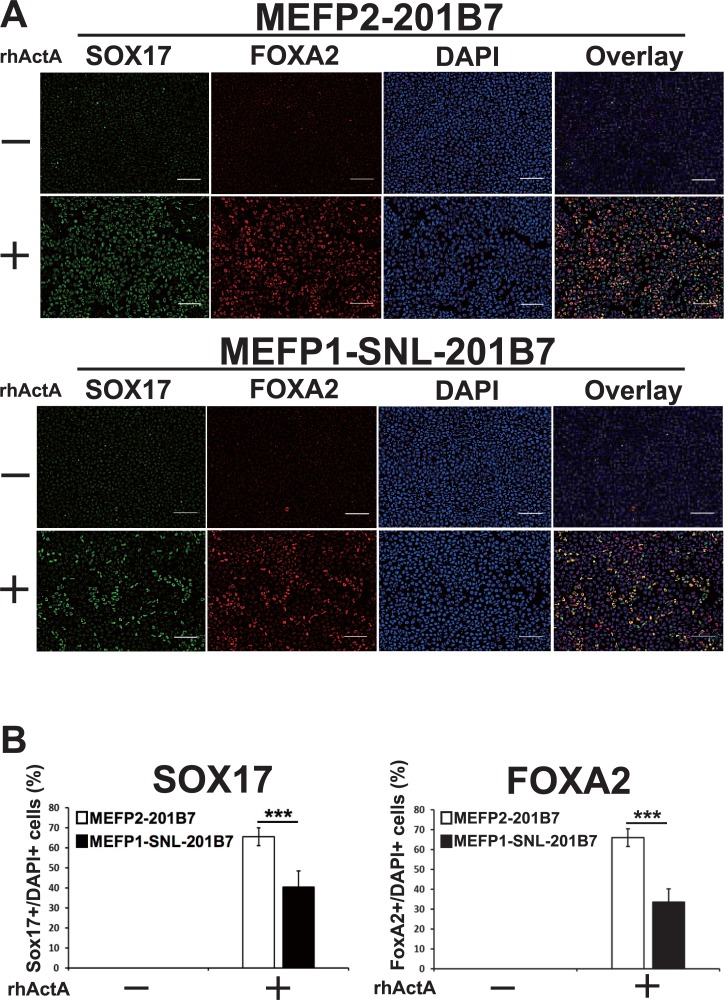
Immunofluorescent staining of SOX17 and FOXA2 proteins in DE differentiated MEFP2- and MEFP1-SNL-201B7 cells. The images of SOX17 (green) and FOXA2 (red), and DAPI (blue) are shown (A). White scale bars, 100 μm. The percentages of SOX17 (B, left panel) and FOXA2 (B, right panel)-positive cells per DAPI-positive cells (n = 8 each) were calculated based on DE marker-positive and DAPI-positive cell numbers. Data represent the mean ± SEM and are representative of three experiments. ***p < 0.001 versus MEFP2-201B7 cells.

**Fig 8:**
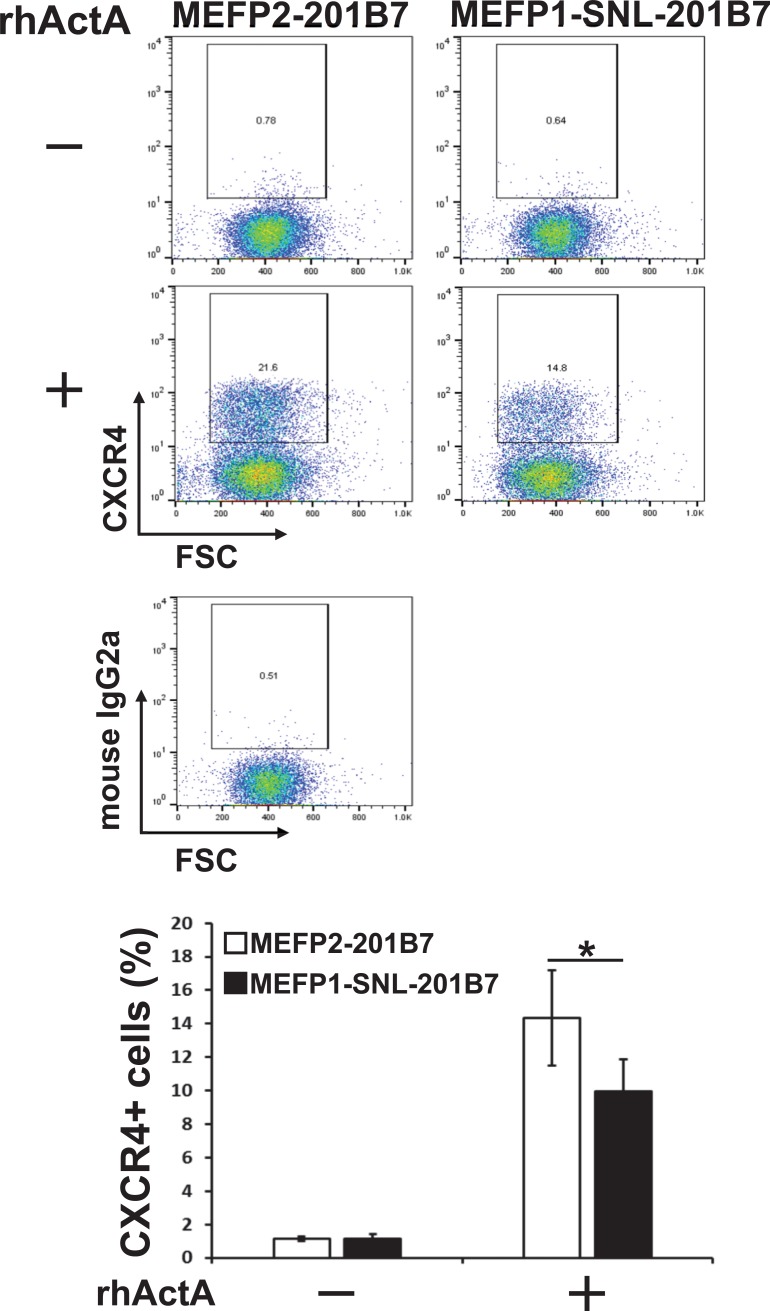
Flow cytometric analysis of CXCR4 expression in DE differentiated MEFP2- and MEFP1-SNL-201B7 cells. The percentages of CXCR4-positive cells (n = 6 each) were analyzed by flow cytometry. Mouse IgG2a antibody was used as an isotype control. Dead cells were excluded using 7-AAD. Data represent the mean ± SEM and are representative of three experiments. *p < 0.05 versus MEFP2-201B7 cells.

Next, to confirm the reduced efficiency of DE differentiation of hiPSCs cultured on SNLs (as described in Fig 1), MEFP1-201B7 colonies were transferred onto MEFs (MEFP2-201B7) and SNLs (MEFP1-SNL-201B7) (Fig 2). We further induced the DE differentiation of these cells using media supplemented with DMSO and rhActA (Fig 2). After a four-day incubation, DE differentiation of the cells was examined by analyzing the expression of *SOX17* and *FOXA2* mRNAs by qPCR (Fig 6), protein by immunofluorescent staining (Fig 7), and CXCR4 by flow cytometry (Fig 8). The expression levels of *SOX17* (Fig 6, left panel) and *FOXA2* (Fig 6, right panel) mRNAs were significantly lower in the DE cells differentiated from MEFP1-SNL-201B7 cells than in the DE cells differentiated from MEFP2-201B7 cells in the presence of rhActA. In contrast, the expression levels of *SOX17* and *FOXA2* mRNAs in the cells differentiated from MEFP2-201B7 and MEFP1-SNL-201B7 cells in the absence of rhActA remained the same before and after the medium change (days 0 or 4) (Fig 6). Photomicrographs of immunofluorescently-stained DE cells differentiated from MEFP2-201B7 or MEFP1-SNL-201B7 cells in the presence and absence of rhActA were analyzed for expressions of SOX17 and FOXA2 protein (Fig 7). There were low numbers of SOX17- and FOXA2-expressing DE cells differentiated from MEFP1-SNL-201B7 cells (Fig 7A, lower panels), and the percentages of these per DAPI-positive DE cells differentiated from MEFP1-SNL-201B7 cells (Fig 7B) were significantly lower than those differentiated from MEFP2-201B7 cells (Fig 7A, upper panels and 7B) in the presence of rhActA. The percentage of CXCR4-positive DE cells differentiated from MEFP1-SNL-201B7 cells was significantly lower than that of CXCR4-positive DE cells differentiated from MEFP2-201B7 cells (Fig 8).

Taken together, these results showed that undifferentiated culture of hiPSCs on SNLs inhibits DE differentiation, suggesting that differences in the murine feeder cell layer used for the maintenance of the undifferentiated state of hiPSCs would affect the efficiency of DE differentiation.

### Differences in the secretion of mouse LIF and ActA proteins by SNLs and MEFs

**Fig 9:**
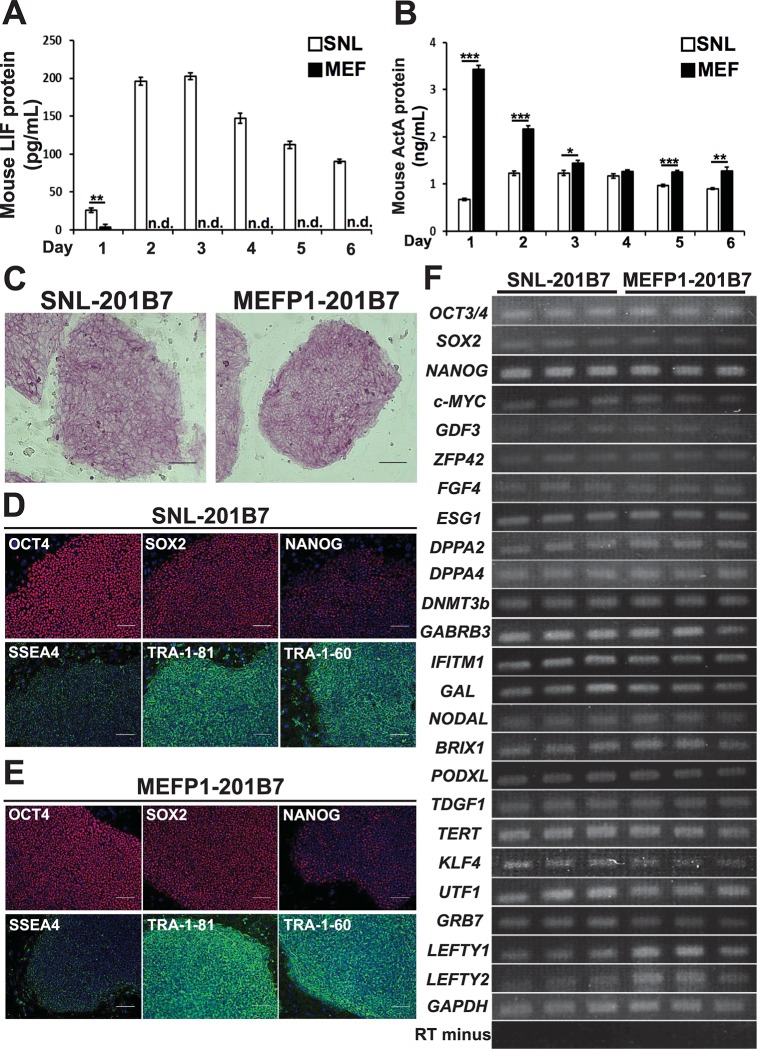
Undifferentiated states of SNL- and MEFP1-201B7 cells. (A and B) Quantification of mouse LIF and ActA protein concentrations in the culture media of SNLs and MEFs. The concentrations of mouse LIF (A) and ActA (B) protein in the collected culture media of SNLs and MEFs from days 1 to 6 (n = 4 each) were measured. Data represent the mean ± SEM. n.d.: not detected. *p < 0.05, **p < 0.01, or ***p < 0.001 versus SNLs. (C) The ALP activities of SNL- or MEFP1-201B7 colonies were analyzed by ALP staining. Black scale bar, 100 μm. (D and E) The expressions of the undifferentiated stem cell marker proteins OCT4, SOX2, SSEA4, TRA-1-81, and TRA-1-60 (red or green) in SNL- (D) or MEFP1-201B7 (E) colonies were analyzed by immunofluorescent staining. Cell nuclei were stained using DAPI (blue). White scale bar, 100 μm. (F) RT-qPCR analysis of undifferentiated stem cell marker gene expression in SNL- and MEFP1-201B7 cells. SNL- and MEFP1-201B7 cells were seeded in Matrigel-coated plates after the removal of feeder cells, and after a 24-hr incubation, total RNA was extracted from these cells and used to synthesize cDNA. The synthesized cDNA was used as a template for PCR. Human *GAPDH*-specific primer and RT-negative sample were used as positive and negative controls, respectively. PCR products were analyzed by agarose electrophoresis and stained with ethidium bromide. Data are representative of three experiments.

Kime *et al*. [23] previously reported that in an analysis of the conditioned medium of SNLs (SNL-CM) and MEFs (MEF-CM), mouse LIF protein is present only in SNL-CM, whereas mouse ActA is enriched in MEF-CM. Thus, to confirm the presence of mouse LIF and ActA proteins in SNL- and MEF-CM used in this study, we measured the concentrations of mouse LIF and ActA proteins in both types of culture medium. A high concentration of mouse LIF protein was present in SNL-CM after 6 days, but not in that of MEF-CM (Fig 9A). In contrast, the concentration of mouse ActA protein was significantly higher in MEF-CM than in SNL-CM (Fig 9B). Thus, mouse LIF protein was enriched in SNL-CM, whereas ActA protein was enriched in MEF-CM.

The activation of the Janus kinase signal transducer (JAK) and signal transducers and activator of transcription 3 (STAT3) (JAK-STAT3) signaling pathways via STAT3 phosphorylation by LIF [24]. In contrast, ActA activates activin/nodal signaling pathways via mothers against decapentaplegic homolog 2 (SMAD2) and/or SMAD3 phosphorylation to maintain the pluripotency of mESCs and hESCs [25–27]. Therefore, to evaluate whether mouse LIF or ActA protein from SNLs and MEFs enhances the activation of each signaling pathway in hiPSCs, the degree of STAT3 and SMAD2 phosphorylation in these hiPSCs was examined by western blotting (S1 Fig). Interestingly, the degree of phosphorylation of STAT3 and SMAD2 proteins was equal in the SNL- and MEFP1-201B7 cells (S1 Fig). Therefore, the LIF and activin signaling pathways of hiPSCs cultured on SNLs and MEFs in rhbFGF-supplemented stem cell medium were equally activated, even though the concentrations of mouse LIF and ActA proteins in the culture medium between SNLs and MEFs differed.

ALP activity and expression of stem cell markers of undifferentiated SNL- and MEFP1-201B7 colonies (seen in Fig 1) was determined [3, 17]. ALP activity was observed in the SNL- (Fig 9C, left panel) and MEFP1-201B7 (Fig 9C, right panel) colonies. The SNL- (Fig 9D) and MEFP1-201B7 (Fig 9E) colonies also expressed marker proteins of undifferentiated stem cells, such as OCT4, SOX2, NANOG, stage-specific embryonic antigen 4 (SSEA4), TRA-1-81, and TRA-1-60.

Next, we examined the expression of the undifferentiated stem cell marker genes in SNL- and MEFP1-201B7 cells prior to changing to DE differentiation-inducing medium. After a 24-hr incubation, total RNA was extracted and the expressions of the marker genes of undifferentiated stem cells [3, 17] in the SNL- and MEFP1-201B7 cells were analyzed (Fig 9F). As shown in Fig 9F, the SNL- and MEFP1-201B7 cells seeded in the Matrigel-coated 96-well plates expressed many of the undifferentiated stem cell marker genes prior to induction of DE differentiation. Thus, the undifferentiated state of the hiPSCs was maintained when they were cultured on either SNLs or MEFs.

### Altered gene-expression of hiPSCs cultured on SNLs and MEFs

Comprehensive RNA sequencing analysis of transcriptome of SNL- and MEFP1-201B7 cells was performed in order to investigate the molecular pathways in hiPSCs affected by their culture on SNLs or MEFs [S5 Table, deposited in the DNA Data Bank of Japan database (accession number DRA006179)]. As shown in Table 1, comprehensive RNA sequencing results showed that mRNA expressions of *LEFTY1*, *LEFTY2*, lysyl oxidase (*LOX*) genes, fibrillin 1 (*FBN1*), v-myb avian myeloblastosis viral oncogene homolog (*MYB*), histone cluster (*HIST*), and histone family genes were up-regulated, while those of chemokine (C-X-C motif) ligand 1 (*CXCL1*), interleukin 23 alpha subunit (*IL23A*), interferon induced transmembrane protein (*IFITM*), and metallothionein genes were down-regulated in MEFP1-201B7 cells in comparison to those of SNL-201B7 cells.

**Table 1:** Focused genes of RNA sequencing analysis, based on ratio of RPKM average between SNL- and MEFP1-201B7 cells.

Gene	RPKM		
	SNL-201B7	MEFP1-201B7	Ratio (MEFP1- per SNL-201B7)
*COL3A1* (collagen, type III, alpha 1)	0.2±0.01	1.0±0.05	4.2
*LOX* (lysyl oxidase)	0.5±0.03	1.2±0.1	2.5
*COL6A3* (collagen, type VI, alpha 3)	0.4±0.03	1.1±0.04	2.5
*HIST1H2BO* (histone cluster 1, H2bo)	1.2±0.2	2.9±0.1	2.4
*BGN* (biglycan)	2.8±0.1	6.5±0.3	2.3
*LOXL1* (lysyl oxidase-like 1)	1.0±0.1	2.2±0.1	2.1
*FBN1* (fibrillin 1)	1.1±0.02	2.3±0.03	2.1
*HIST1H4K* (histone cluster 1, H4k)	2.4±0.2	5.1±0.4	2.1
*HIST1H2AC* (histone cluster 1, H2ac)	2.7±0.3	5.5±0.3	2.1
***LEFTY2* (left-right determination factor 2)**	9.6±0.3	18.7±0.4	2.0
*HIST1H4J* (histone cluster 1, H4j)	2.5±0.2	5.1±0.4	2.0
*HIST3H2A* (histone cluster 3, H2a)	3.6±0.2	6.4±0.3	1.8
*OTX2* (orthodenticle homeobox 2)	6.5±0.3	11.2±0.2	1.7
*MYB* (v-myb oncogene homolog)	0.6±0.02	1.0±0.05	1.7
*HIST1H2BN* (histone cluster 1, H2bn)	2.4±0.2	3.8±0.1	1.6
*HIST1H2BD* (histone cluster 1, H2bd)	2.0±0.2	3.3±0.2	1.6
*HIST1H2BJ* (histone cluster 1, H2bj)	2.1±0.1	3.0±0.1	1.5
***LEFTY1* (left-right determination factor 1)**	16.9±0.6	24.4±0.9	1.4
*H1F0* (H1 histone family, member 0)	16.5±0.4	23.0±0.3	1.4
*H2AFJ* (H2A histone family, member J)	4.4±0.1	6.1±0.1	1.4
***XIST* (X inactive specific transcript)**	0.037±0.003	0.048±0.003	1.3
*IFITM1* (interferon induced transmembrane protein 1)	244.1±3.0	165.7±2.8	0.7
*IFITM3* (interferon induced transmembrane protein 3)	241.1±3.1	163.9±2.2	0.7
*FBN2* (fibrillin 2)	10.8±0.2	7.2±0.1	0.7
*MT2A* (metallothionein 2A)	149.3±3.0	106.4±1.6	0.7
*MT1G* (metallothionein 1G)	142.5±2.6	92.8±1.2	0.7
*MT1F* (metallothionein 1F)	29.8±0.8	21.2±0.5	0.7
*MT1L* (metallothionein 1L (gene/pseudogene))	3.8±0.2	2.7±0.3	0.7
*MT1A* (metallothionein 1A)	4.5±0.2	2.7±0.1	0.6
*MT1E* (metallothionein 1E)	76.1±1.1	44.3±1.1	0.6
*MT1H* (metallothionein 1H)	23.0±0.9	13.9±0.5	0.6
*MT1M* (metallothionein 1M)	11.2±0.5	7.0±0.5	0.6
*MT1JP* (metallothionein 1J, pseudogene)	7.4±0.4	4.1±0.3	0.6
*CXCL1* (chemokine (C-X-C motif) ligand 1)	4.6±0.2	2.5±0.2	0.5
*IL23A* (interleukin 23, alpha subunit p19)	2.4±0.2	1.3±0.1	0.5
*GBX2* (gastrulation brain homeobox 2)	1.6±0.1	0.7±0.1	0.4
*TTN* (titin)	1.4±0.03	0.6±0.02	0.4
*GAPDH* (glyceraldehyde-3-phosphate dehydrogenase)	1587.7±14.3	1789.9±13.0	

From RNA sequencing transcriptome data (S1 Table) deposited in the DNA Data Bank of Japan database (accession number DRA006179), we identified genes with ratios of > 1.3 or < 0.7, based on the ratio of RPKM average between SNL- and MEFP1-201B7 cells (n = 6 each). Data represent the mean ± the standard error of the mean.

**Fig 10:**
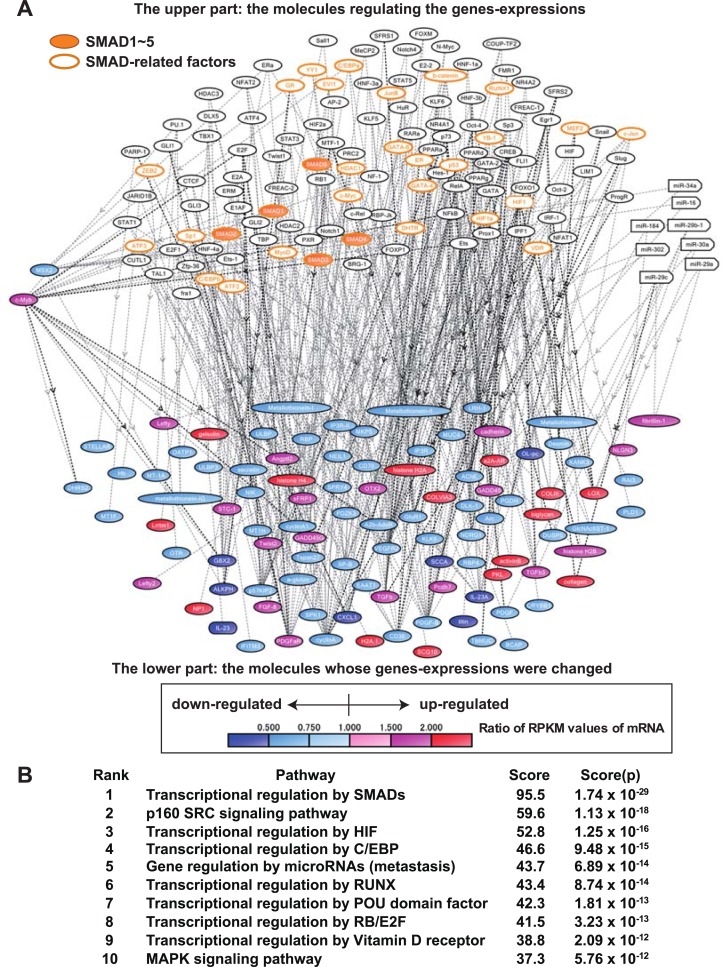
Molecular network analysis of SNL- and MEFP1-201B7 cells by KeyMolnet program using the comprehensive RNA sequencing data. Using the comprehensive RNA sequencing data (deposited in the DNA Data Bank of Japan database (accession number DRA006179) and summarized in Table 1) of SNL-201B7 and MEFP1-201B7 (n = 6 each), the molecular networks, pathways, and transcriptional regulators of altered gene-expression were analyzed by the KeyMolnet program. (A) Molecular network of the supposed transcription factors and the regulated genes of SNL- and MEFP1-201B7 cells. The upper part shows the molecules that regulate gene expression. The orange -filled and -framed circles in the upper part indicate SMAD1-5 and SMAD-related factors, respectively. The lower part shows those molecules whose gene expression was altered. The ratio of the reads per kilobase per million mapped reads (RPKM) values of expressed mRNA of MEFP1-201B7 cells, against those of SNL-201B7 cells, revealed by the comprehensive RNA analysis, are shown in red and blue colors that indicate up- and down-regulated molecules, respectively. (B) The supposed molecular pathways and their calculated scores in the altered genes-expression of SNL- and MEFP1-201B7 cells. The extracted molecular network shown in the panel (A) was compared with distinct canonical pathways of the KeyMolnet library. This makes it possible to identify the canonical pathway showing the most significant contribution to the extracted network. The score and score (p) were calculated as described in the experimental procedures. In the list of (B), several abbreviations were used, indicated below. SRC: steroid receptor coactivator, HIF: hypoxia-inducible factor, C/EBP: CCAAT-enhancer-binding protein, RUNX: Runt-related transcription factor, POU: Pit-1, Octamer transcription factor and Unc-86, RB: Retinoblastoma gene product, and MAPK: MAP kinase.

Next, transcriptional regulators were predicted by *in silico* molecular network analysis with KeyMolnet software using the comprehensive RNA sequencing results. This analysis showed that the up-regulation of mRNA expressions of histone *H4*, *H2A* cluster genes, and *c-MYB* genes and down-regulation of metallothionein 1 and 2 genes in MEFP1-201B7 cells were related to the regulation of SMAD1-5 and SMAD-related factors and microRNAs, such as miR-16, -23, -30, -34, -184, and -302, acted as regulatory molecules of gene expression altered in SNL-201B7 and MEFP1-201B7 cells (Fig 10A). KeyMolnet analysis also showed that transcriptional regulation by SMAD, p160 SRC signaling pathway, and transcriptional regulation of HIF were highly scored (Fig 10B). Therefore, these results suggested that these defined-signaling pathways are highly involved in gene expression changes in hiPSCs cultured on SNLs and MEFs.

### Up-regulated expression of *hXIST exon 4* in hiPSCs cultured on MEFs compared with that of SNLs

**Fig 11:**
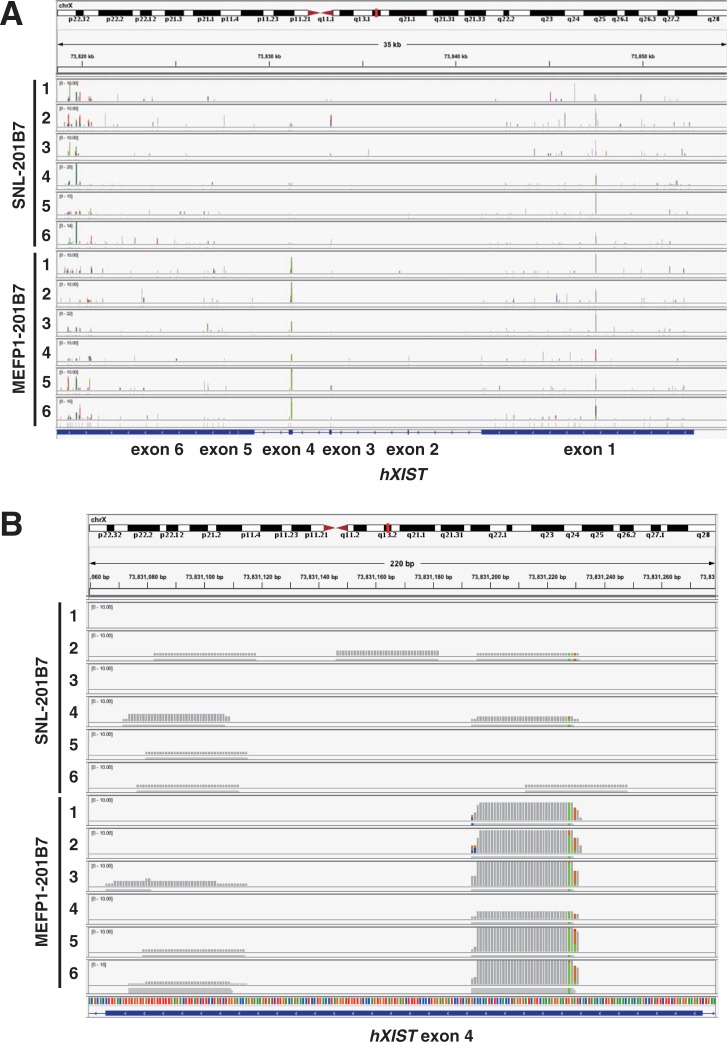
Mapping of RNA-sequencing of SNL-201B7 and MEFP1-201B7 on the human genome, hg38. (A) RNA-sequencing of SNL-201B7 and MEFP1-201B7 (n = 6, each) was mapped on human genome, hg38, especially around the *hXIST* gene. The result was visualized by genome viewer (IGV_2.4.4). The vertical axis indicates number of reads of RNA-sequencing. (B) Mapping of RNA-sequencing on *hXIST* exon 4. The specific, restricted RNA-fragment was found to be expressed in MEFP1-201B7 at levels higher than in SNL-201B7.

**Fig 12:**
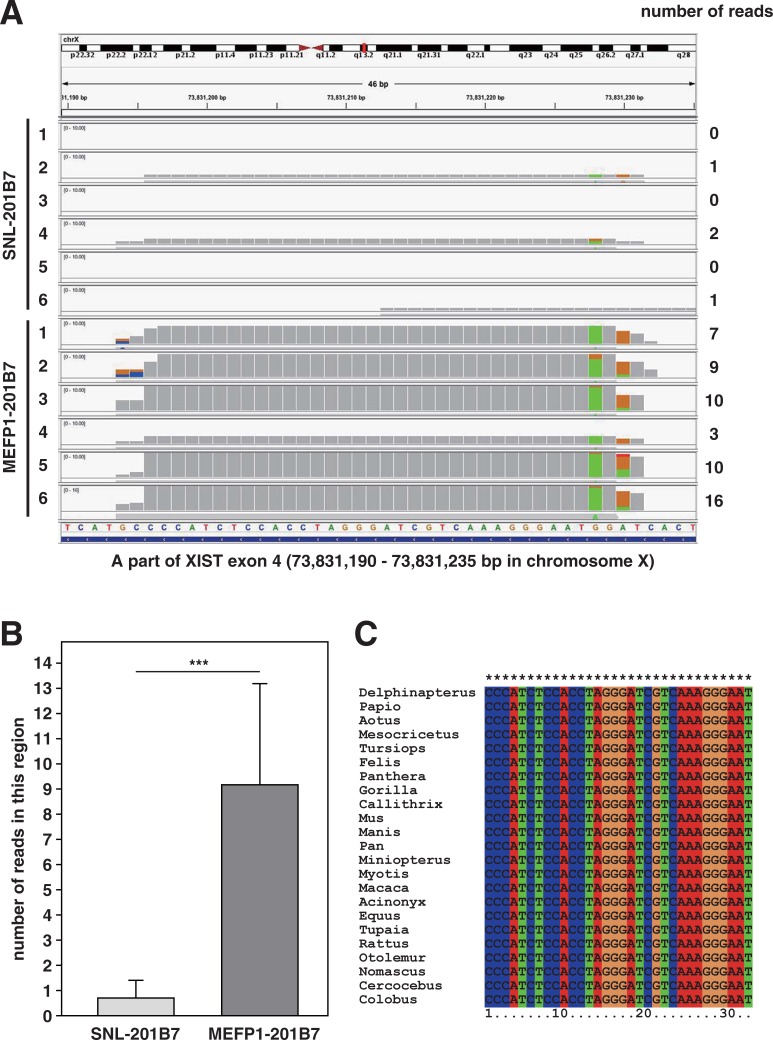
Approximately 32-nucleotide conserved RNA fragment in *hXIST* exon4 highly expressed in MEFP1-201B7. (A) Discovery of the 32 nucleotides RNA sequence of *hXIST* exon 4 and the numbers of reads of RNA-sequencing in this region (n = 6). (B) The comparison of the average numbers of reads of RNA-sequencing in this region. (C) Complete conservation of the 32-nucleotide sequence in *hXIST* exon 4 among mammals (human, dolphin, bat, monkey, cat, mouse, etc.). 32-nucleotide sequence was searched in non-redundant nucleotide sequences NCBI database by BLAST program. The alignment is shown as DNA sequences, visualized by ClustalX software. *Delphinapterus*: *Delphinapterus leucas* (XR_002645620.1); *Papio*: *Papio anubis* (XR_641986.3); *Aotus*: *Aotus nancymaae* (XR_001111121.2); *Mesocricetus*: *Mesocricetus auratus* (XR_002381491.1); *Tursiops*: *Tursiops truncatus* (XR_002175793.1); *Felis*: *Felis catus* (XR_002152615.1); *Panthera*: *Panthera pardus* (XR_002076944.1); *Gorilla*: *Gorilla gorilla gorilla* (XR_002004498.1); *Callithrix*: *Callithrix jacchus* (XR_001909167.1); *Mus*: *Mus musculus* (AH003266.2); *Manis*: *Manis javanica* (XR_001851889.1); *Pan*: *Pan troglodytes* (XR_676711.2); *Miniopterus*: *Miniopterus natalensis* (XR_001603751.1); *Myotis*: *Myotis davidii* (XR_435573.2); *Macaca*: *Macaca fascicularis* (XR_287323.2); *Acinonyx*: *Acinonyx jubatus* (XR_001431630.1); *Equus*: *Equus caballus* (XR_291129.2); *Tupaia*: *Tupaia chinensis* (XR_337298.2); *Rattus*: *Rattus norvegicus* (NR_132635.1); *Otolemur*: *Otolemur garnettii* (XR_001161860.1); *Nomascus*: *Nomascus leucogenys* (XR_122040.3); *Cercocebus*: *Cercocebus atys* (XR_001010237.1); *Colobus*: *Colobus angolensis palliatus* (XR_001000513.1).

**Fig 13:**
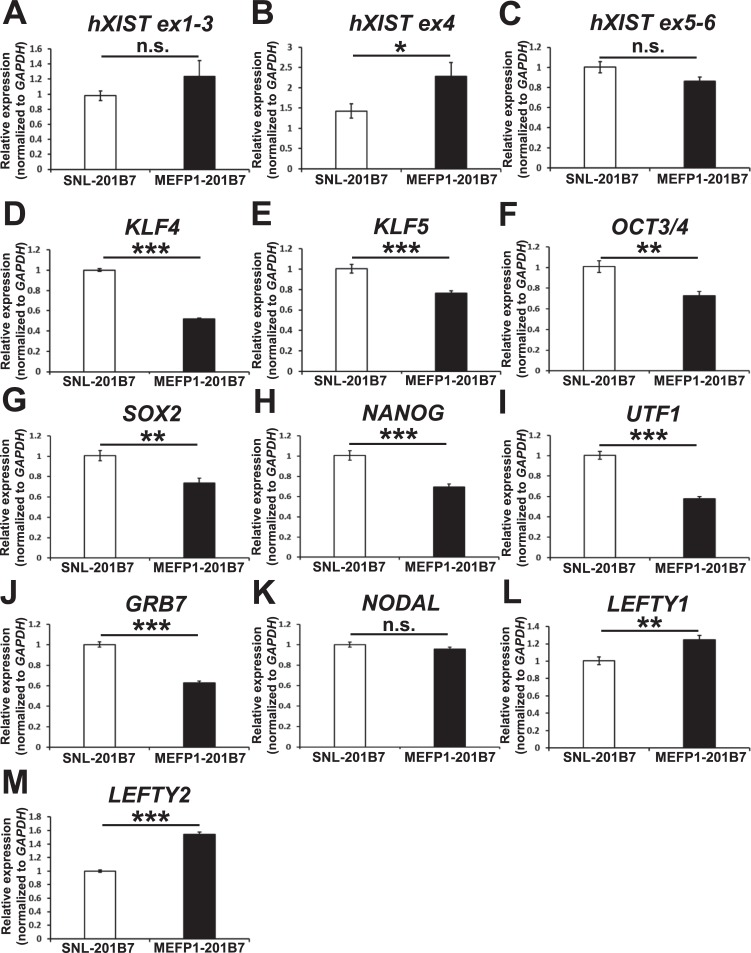
Changes in gene-expression of *hXIST* exons and of the undifferentiated stem cell marker genes of SNL- and MEFP1-201B7 cells. (A–J) RT-qPCR analysis was used to evaluate the mRNA expression of hXIST exons or undifferentiated stem cell marker genes in SNL- and MEFP1-201B7 cells. Total RNA of SNL- and MEFP1-201B7 cells was used for cDNA synthesis. The relative mRNA expression levels of *hXIST* exons 1–3 (*ex1-3*) (A), exon 4 (*ex4*) (B), and exons 5–6 (*ex5-6*) (C) (n = 12 each) or the undifferentiated stem cell markers *KLF4* (D), *KLF5* (E), *OCT3/4* (F), *SOX2* (G), *NANOG* (H), *UTF1* (I), *GRB7* (J), *NODAL* (K), *LEFTY1* (L), and *LEFTY2* (M) (n = 6 each) were determined by RT-qPCR, normalized to that of human *GAPDH*, and expressed in relation to the levels in SNL-201B7 cells (set as 1). Data represent the mean ± SEM and are representative of two or three experiments. n.d.: not detected. *p < 0.05, **p < 0.01, or ***p < 0.001 versus SNL-201B7 cells. n.s.: not significant.

Interestingly, the expression of non-coding h*XIST*, which plays the major role in X chromosome inactivation [15, 16], was up-regulated in MEFP1-201B7 cells, in comparison to that in SNL-201B7 cells (Table 1). *hXIST* has approximately 17 kb of cDNA sequences across eight different exons [16], and X chromosome inactivation occurs as a result of the protein complexes that interact with *XIST* RNA. Tomoda *et al*. [10] reported that *XIST* expression was down-regulated by cells cultured on SNLs or by rhLIF supplementation in XaXi hiPSCs. Cells cultured on MEFs would up-regulate *XIST* expression in hiPSCs. Thus, we examined the exons of *hXIST* expression in SNL- and MEFP1-201B7 cells in the results of comprehensive RNA sequencing analysis (Fig 11A) and found that the *hXIST exon 4* (*hXIST ex4*) in MEFP1-201B7 cells was up-regulated compared with that of SNL-201B7 cells (Fig 11A). This up-regulation in MEFP1-201B7 cells occurred at the region of *hXIST ex4* (Fig 11B), especially at the specific approximately 32-nucleotide sequence of *hXIST ex4* (Fig 12A). The number of reads of RNA-sequencing of MEFP1-201B7 was approximately ten times that of SNL-201B7 (Fig 12B). This sequence was completely conserved among a wide variety of mammals (Fig 12C) as previously reported [28], indicating its functional importance. In addition, using qPCR, we confirmed the up-regulation of gene-expression of *hXIST ex4* in MEFP1-201B7 cells, but not in *hXIST ex1-3* nor *ex5-6*, compared with SNL-201B7 cells (Fig 13A–13C).

### Differences in expression of stem cell marker genes between hiPSCs cultured on SNLs and MEFs

As shown in Fig 9F and Table 1, SNL-201B7 cells expressed high levels of *KLF4*, *UTF1*, and *GRB7* transcripts, but low levels of *LEFTY1* and *LEFTY2* transcripts compared to MEFP1-201B7 cells. SNLs stably express LIF, and LIF regulates the expression of *Klf4* and *Klf5* in mESCs [29]. KLF4 directly interacts with OCT4 and SOX2 for reprogramming mESCs and iPSCs [30], and KLF4 and KLF5 regulate *NANOG* expression in hESCs [31, 32]. Aksoy *et al*. [33] reported that *Klf4* and *Klf5* inhibit mesoderm and endoderm differentiation in mESCs, respectively. In addition, Hanawa *et al*. showed that *LEFTY1* knockdown inhibits DE differentiation in hiPSCs [34]. In contrast, ActA induces NANOG expression via activation of activin/nodal signaling pathways, and inhibition of this signaling pathway downregulates the expression of *LEFTY1*, *LEFTY2*, and *NODAL*, located downstream of transforming growth factor-β (*TGF-β*), ActA, or nodal signaling pathways, in hESCs [25]. Therefore, differences in the expression of these genes between hiPSCs cultured on SNLs and MEFs may affect DE differentiation. Here, we analyzed the mRNA expression levels of *KLF4*, *KLF5*, *OCT3/4*, *SOX2*, *NANOG*, *UTF1*, *GRB7*, *LEFTY1*, and *LEFTY2* in SNL- and MEFP1-201B7 cells. The total RNA samples extracted for the work shown in Fig 9F were also used in this qPCR analysis. When compared with MEFP1-201B7 cells, the mRNA expression levels of *KLF4*, *KLF5*, *OCT3/4*, *SOX2*, *NANOG*, *UTF1*, and *GRB7* (Fig 13D–13J) were significantly upregulated in SNL-201B7 cells, whereas the expression levels of *NODAL* (Fig 13K) remained unchanged. Interestingly, the mRNA expression levels of *LEFTY1* and *LEFTY2* (Fig 13L and 13M) were significantly upregulated in MEFP1-201B7 cells when compared with that in SNL-201B7 cells.

Taken together, these results showed that differences in the culture conditions of SNLs and MEFs used for the maintenance of the undifferentiated state of hiPSCs, alter the expression of pluripotency-related genes by the defined-signaling pathways and affect DE differentiation of hiPSCs.

## Discussion

PSCs occur in two different states, naïve and primed. The maintenance of naïve mouse PSCs, such as ESCs from the blastocyst [9], requires the activation of LIF/bone morphogenetic protein signaling, and naïve PSCs can be converted into murine primed cells, such as epiblast stem cells (EpiSCs) from the pre-gastrulation or early gastrulation embryo, by incubation in ActA- and bFGF-containing medium [23, 35]. hESCs are isolated from human blastocysts and have similar molecular features to naïve mESCs [9]. However, maintenance of both hESCs and hiPSCs requires the activation of bFGF and TGF-β or activin signaling pathways, but not LIF signaling [36]. hESCs and hiPSCs also share many characteristics of primed pluripotent cells, such as low expression levels of the naïve pluripotency marker *NANOG* [37]. In addition, differences in the culture conditions for maintenance of pluripotency in female mouse and human ESCs or iPSCs affect X chromosome activity status [10, 23]. Female hiPSCs cultured on SNLs have XaXa, whereas female hiPSCs cultured on MEFs or human fibroblasts and hiPSCs cultured on SNLs at an early passage (such as passage five) have XaXi [10]. These XaXi hiPSCs are down-regulated in *XIST* expression through culture on rhLIF-supplemented medium or by being passaged more than 15 times on SNLs and then can be converted to XaXa hiPSCs [10]. In addition, one of the X chromosomes of XaXa female hiPSCs is inactivated by endothelial cell differentiation [10]. In this study, we confirmed high concentrations of mouse LIF and ActA proteins in the culture media of SNLs and MEFs, respectively (Fig 9A and 9B). Colonies of female hiPSC lines 201B7 and 253G1 passaged more than 20 times on SNLs were transferred onto SNLs and MEFs in rhbFGF-supplemented stem cell medium to prepare the SNL- and MEFP1-201B7 or -253G1 cells (Fig 1). We demonstrated that *hXIST ex4* was up-regulated in hiPSCs cultured on MEFs in comparison to that in SNL-201B7 cells (Table 1 and Figs 11, 12 and 13B), suggesting that the XaXa status of hiPSCs cultured on SNLs would be changed to XaXi status by culture on MEFs. We showed that the transcriptional regulation by SMADs, p160 SRC signaling pathways, and transcriptional regulation of HIF were highly involved in the gene expression changes in SNL- and MEFP1-201B7 cells (Fig 10) and also found that the mRNA expression levels of the stem cell markers *KLF4*, *KLF5*, and *NANOG* in SNL-201B7 cells were decreased after being passaged on MEFs (Fig 13). We further demonstrated that the efficiency of DE differentiation from 201B7 and 253G1 cells cultured on SNLs was reduced when compared with those cultured on MEFs (Figs 1–8). Therefore, female hiPSCs that have been passaged more than 20 times on SNLs in rhbFGF-supplemented stem cell medium might be converted to the naïve pluripotent state via a XaXa status change; these hiPSCs would then be converted to the XaXi state via the up-regulation of *XIST* expression when transferred onto MEFs by these defined-signaling pathways. This conversion would affect the efficiency of DE differentiation.

Tomoda *et al*. [10] reported that the expression ratio between X-linked genes and autosomal genes of only the male hESCs and hiPSCs but not female XX hiPSCs, cultured on SNLs, were similar to those of the deposited microarray data sets of XaXi cell lines. Ojala *et al*. [4] showed that the male hiPSC line UTA.00525.LQT2 cultured on MEFs had increased cardiomyocyte differentiation compared to that cultured on SNLs or Matrigel, but the cardiomyocyte differentiation in the male hiPSC line UTA.00106.hFF cultured on MEFs was decreased compared to that of cells cultured on SNLs. Male hiPSC lines, such as Tic or dotcom, were maintained when grown on MEFs in 10 ng/ml bFGF-supplemented stem cell medium and then efficiently differentiated into hepatocytes or enterocyte-like cells of the small intestine via DE differentiation [2, 38, 39]. These reports suggest that, compared to female hiPSCs, male hiPSCs cultured on SNLs or MEFs may possess the characteristics of XaXi hiPSCs and efficiently differentiate into mesoderm or DE-related cells.

Activation of the LIF/STAT3 signaling pathway via STAT3 phosphorylation upregulates the target gene products through STAT3 and/or Nanog cooperation, thereby inhibiting the differentiation of mesoderm and endoderm in mESCs [29, 40]. In addition, Aksoy *et al*. [33] reported that *Klf4* and *Klf5* upregulated by LIF/STAT3 signaling in mESCs inhibit mesoderm and endoderm differentiation, respectively. These genes regulate the expression of *Nanog* and contribute to the inhibition of endoderm differentiation in mESCs [41–43]. We showed that the mRNA expression levels of *KLF4*, *KLF5*, and *NANOG* were upregulated in 201B7 cells cultured on SNLs (Fig 13D, 13E and 13H) and that the efficiency of DE differentiation was reduced compared to 201B7 cells cultured on MEFs (Figs 1–8). Therefore, the induction of DE differentiation from hiPSCs may be inhibited by the expression of *KLF4*, *KLF5*, and *NANOG* genes upregulated in the cells stably expressing mouse LIF protein secreted from SNLs. Interestingly, the degree of phosphorylation of STAT3 protein was equal in the SNL- and MEFP1-201B7 cells (S1 Fig). Therefore, the other signaling pathways, such as p160 SRC signaling or transcriptional regulation by HIF in Fig 10B, may be involved in the differences of these pluripotent-related genes expression. Furthermore, the core pluripotency factors OCT3/4, SOX2, and NANOG as well as UTF1 and GRB7 are upregulated and promote cellular reprogramming toward pluripotency in 201B7 cells cultured on SNLs compared with MEFs (Fig 13F, 13G, 13I and 13J) [3, 17]. *UTF1* is expressed in the primitive ectoderm and is downregulated at the early streak stage and is transcriptionally regulated by OCT4 and SOX2 [44]. Thus, *UTF1* upregulation contributes to reprogramming and pluripotency maintenance. Taken together, the stable secretion of mouse LIF protein upregulates these genes in 201B7 cells cultured on SNLs thereby maintaining their pluripotency but weakening the gene expression levels responsible for induction of DE differentiation when compared to that of hiPSCs cultured on MEFs.

Interestingly, we showed that the degree of phosphorylation of SMAD2 protein was equal in the SNL- and MEFP1-201B7 cells (S1 Fig), and the transcriptional regulation by SMAD was highly scored by *in silico* molecular network analysis with KeyMolnet software using the comprehensive RNA sequencing results (Fig 10B). We also found that the expression levels of *LEFTY1* and *LEFTY2* mRNAs were upregulated in 201B7 cells cultured on MEFs compared to those cultured on SNLs (Table 1 and Fig 13L and 13M). In the mouse blastocyst, *Lefty* is the stemness marker and is highly expressed in the inner cell mass and trophectoderm [3, 17, 45]. *Lefty* expression is regulated by cooperation of KLF4, OCT4, and SOX2 in mESCs [46]. Sekkai *et al*. [47] and Dvash *et al*. [48] reported that *Lefty* expression increases in the LIF-free culture conditions of mESCs or during differentiation of hESCs to an embryoid body. ESC differentiation by retinoic acid also increases *Lefty* expression in mouse embryonal carcinoma cells [49]. Thus, these reports suggest that *Lefty* expression regulates self-renewal as well as acts as the gate that leads to ESC differentiation. In addition, Kim *et al*. [50] reported that suppression of *Lefty1* expression enhances self-renewal and increases differentiation potential, whereas suppression of *Lefty2* enhances self-renewal but reduces differentiation potential in mESCs under the conditions employed for differentiation. Teo *et al*. [51] reported that activin A activates SMAD2/3 to promote definitive endoderm differentiation through activation of receptor-like kinase 4/5/7 and that *Lefty* is directly regulated by SMAD2/3 [52]. Hanawa *et al*. [34] recently reported that hepatocyte nuclear 4 alpha knockdown inhibits the mRNA expression of *FOXA2*, *SOX17*, and *LEFTY1* and that *LEFTY1* knockdown also inhibits the mRNA expression of *Goosecoid*, *SOX17*, and transcription factor *GATA-4* in the male hiPSC line Tic, suggesting that *LEFTY1* promotes DE differentiation in hiPSCs. Therefore, the upregulation of *LEFTY1* and *LEFTY2* expression by activin A signaling pathways via transcriptional regulation of the other SMAD proteins but not SMAD2 or the other signaling pathways defined in Fig 10B in hiPSCs cultured on MEFs may help to promote DE differentiation.

By comprehensive RNA sequencing analysis, we found that *HIST*, histone family genes, *XIST*, *H2A* and *H4* genes were up-regulated in MEFP1-201B7 cells in comparison to those of SNL-201B7 cells (Table 1 and Fig 10A). In addition, *hXIST ex4* expression in MEFP1-201B7 cells was up-regulated in comparison to SNL-201B7 cells (Table 1 and Figs 11, 12 and 13B). Histone octamers composed of two core histone proteins, H4-3 and H2A-H2B, form heterodimers and package the DNA double helix, resulting in the formation of chromatin. The chromatin undergoes further condensation to form the chromosome. X chromosome inactivation is established by the protein complexes that interact with XIST RNA. Capparros et al. [28] reported that exon 4 of *XIST* RNA is highly conserved at the primary sequence level and is predicted to form a stable stem-loop structure. We also predicted the secondary structure of our identified 32-nucleotide RNA (S2 Fig), which forms stem-like structures, thus, there may be some molecules interacting with this short RNA. Although the deletion of *Xist exon 4* in mouse did not cause detectable effects on X inactivation, this mutant reduced *Xist* RNA expression to the steady-state level, suggesting that the deletion affects transcription or processing of *Xist* RNA [28]. In mammalian cells, the *XIST* RNA recruits polycomb repressor complex (PRC) 1 and PRC2 to the process of X chromosome inactivation [53, 54]. PRC1 and PRC 2 catalyze the mono-ubiquitylation of H2A lysine 119 and methylation of H3 lysine 27, respectively [55]. Therefore, the up-regulation of *hXIST ex4* and *H2A* may promote X chromosome inactivation by inducing the mono-ubiquitylation H2A of PRC1.

Using molecular network analysis on our comprehensive RNA sequencing results, we showed that microRNAs, such as miR-34 and miR-302, function as regulatory molecules of gene expression in SNL-201B7 and MEFP1-201B7 cells (Fig 10). Among these miRNAs, miR-34 inhibits reprogramming by repression of pluripotent genes, including *Nanog*, *Sox2*, and *N-Myc* [56], while miR-302 regulates Brg1 chromatin remodeling complex composition in hESCs via direct repression of the Brg1-associated factor (BAF) 53a and BAF170 [57]. The repression of BAF170 by miR-302 upregulates miR-302 and induces the differentiation of mesendoderm and definitive endodermal progenitor cells in hESCs [57]. Therefore, these miRNAs, particularly miR-34 and miR-302, may promote the DE differentiation and regulate transcriptional gene expression in hiPSCs cultured on SNLs or MEFs.

Recently, the maintenance of undifferentiated state of the hiPSCs is required to the feeder free-culture condition for clinical application, such as hiPSCs-derived differentiated cell transplantation for the regenerative therapy. Various feeder-free culture systems for hiPSCs have been developed [58–61]. However, when hiPSCs-derived differentiated cells are used for *in vitro* disease model and drug screening for therapeutical development, the feeder-free culture condition would not be necessarily needed for hiPSCs maintenance. Thus, in these cases, it is important to develop the proper culture system of hiPSCs maintenance for obtaining a large number of hiPSCs-derived differentiated cells. Ojala *et al*. [4] found that culturing on SNLs and MEFs promoted cardiac differentiation of hESCs and hiPSCs and inhibited ectoderm-derived neuronal differentiation when compared with feeder-free culture conditions. Therefore, our finding in this study would contribute the development of hiPSCs maintenance for obtaining a large number of hepatic, pancreatic or intestinal cells, DE lineage cells.

## Conclusion

**Fig 14:**
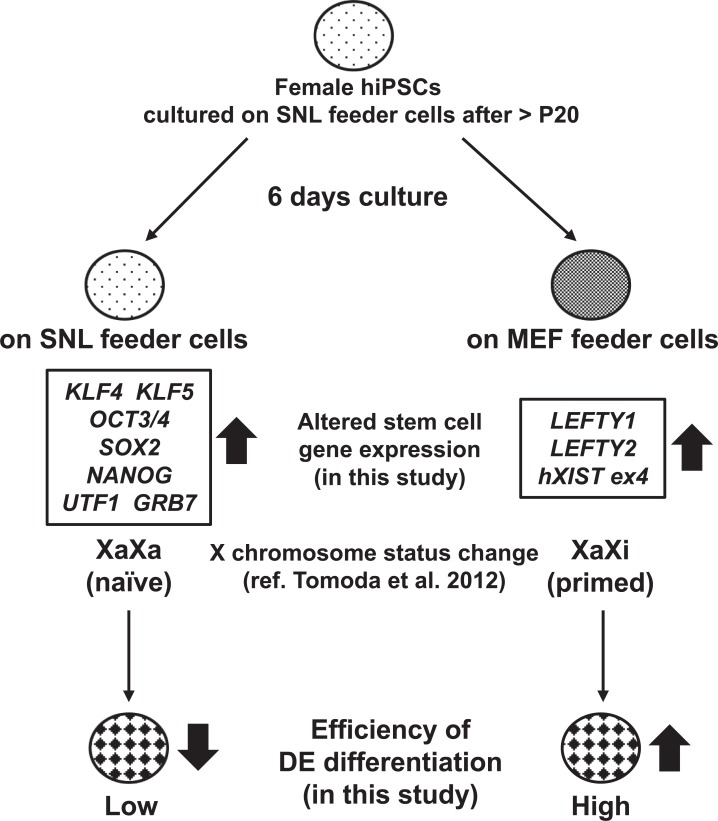
Schematic summary of this study. This scheme includes the results and conclusions of the present study [10].

Based on our findings and those of previous reports and as shown in Fig 14, we suggest that differences in the culture conditions of SNLs and MEFs for maintenance of the undifferentiated state of hiPSCs alter the expression of pluripotency-related genes and affect the X chromosome active/inactive status, thereby affecting the efficiency of DE differentiation from hiPSCs.

## Supporting information

S1 Fig Signaling pathways of hiPSCs activated by mouse LIF or ActA in the culture medium of SNL or MEF feeder cells. Western blotting of SNL- and MEFP1-201B7 cells for the detection of STAT3, phosphorylated STAT3 (pSTAT3), SMAD2, phosphorylated SMAD2 (pSMAD2), and β-actin protein. The levels of STAT3 and SMAD2 phosphorylation were normalized to those of STAT3 and SMAD2 proteins (n = 9 each). pSTAT3/STAT3 and pSMAD2/SMAD2 protein levels were expressed relative to those of the SNL-201B7 cells (set as 1) (n = 9 each). Data are presented as the mean ± SEM of three independent experiments. The results were reproducible. n.s.: not significant.
(PDF)

S2 Fig Predicted secondary structure of the conserved 32 nucleotides RNA in *hXIST exon 4*. The secondary structure of the conserved 32 nucleotides RNA in *hXIST* RNA was predicted using the RNAfold web server (http://rna.tbi.univie.ac.at/cgi-bin/RNAWebSuite/RNAfold.cgi). The secondary structures are colored by base-pair probabilities (A) and by positional entropy (B). RNA parameters are described in Supplementary reference 2.
(PDF)

S1 Table Marker gene-specific primers used in qPCR for undifferentiated human cells, definitive endoderm and human X inactive specific transcript exon RNAs. (XLSX)

S2 Table Primary or secondary antibodies used in the immunofluorescent staining. (XLSX)

S3 Table Undifferentiated human stem cell marker-gene specific primers used in RT-PCR. (XLSX)

S4 Table Statics and histogram analyses of RNA sequences of SNL- and MEFP1-201B7 cells. The p-values of the normalized RPKM values of RNA sequences of SNL- and MEFP1-201B7 cells (n = 6 each) were calculated. The histogram was generated and is shown at the bottom of the Table.
(XLSX)

S5 Table Comprehensive RNA sequencing analysis of SNL- and MEFP1-201B7 cells. SNL- or MEFP1-201B7 cells (n = 6 each) were seeded in Matrigel-coated 96-well plates after the removal of feeder cells. After incubation for 24 hr, total RNA was extracted and used for RNA sequencing transcriptome analysis. The reads per kilobase per million mapped reads (RPKM) were calculated for the mRNA transcripts in Refseq database. The ratio of each gene in Refseq database was calculated using RPKM averages in SNL- and MEFP1-201B7 cells.
(XLSX)
