# Supplementary figures and images for: Different murine-derived feeder cells alter the definitive endoderm differentiation of human induced pluripotent stem cells
Source: PLoS One. 2018 Jul 26;13(7):e0201239. doi: 10.1371/journal.pone.0201239 (PMC6062072; doi:10.1371/journal.pone.0201239)

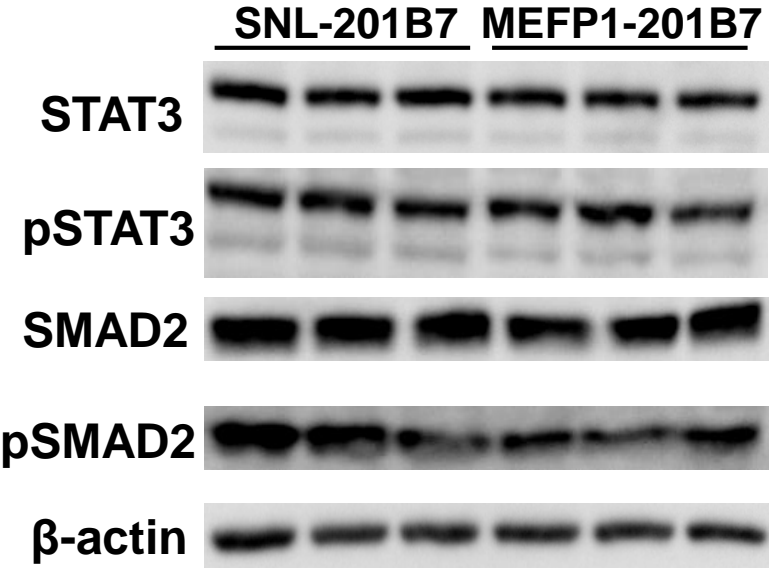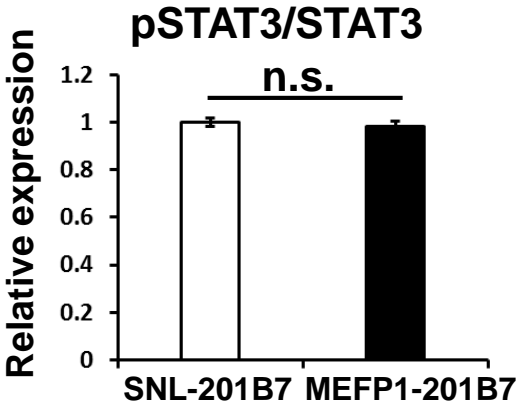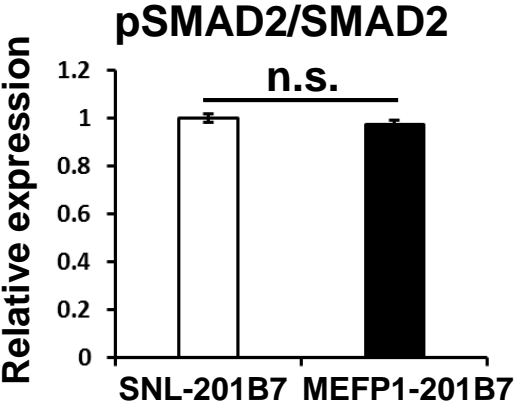

Supplement: S1 Fig — Western blotting of SNL- and MEFP1-201B7 cells for the detection of STAT3, phosphorylated STAT3 (pSTAT3), SMAD2, phosphorylated SMAD2 (pSMAD2), and β-actin protein. The levels of STAT3 and SMAD2 phosphorylation were normalized to those of STAT3 and SMAD2 proteins (n = 9 each). pSTAT3/STAT3 and pSMAD2/SMAD2 protein levels were expressed relative to those of the SNL-201B7 cells (set as 1) (n = 9 each). Data are presented as the mean ± SEM of three independent experiments. The results were reproducible. n.s.: not significant. (PDF) [file pone.0201239.s001.pdf]

Figure S2

A

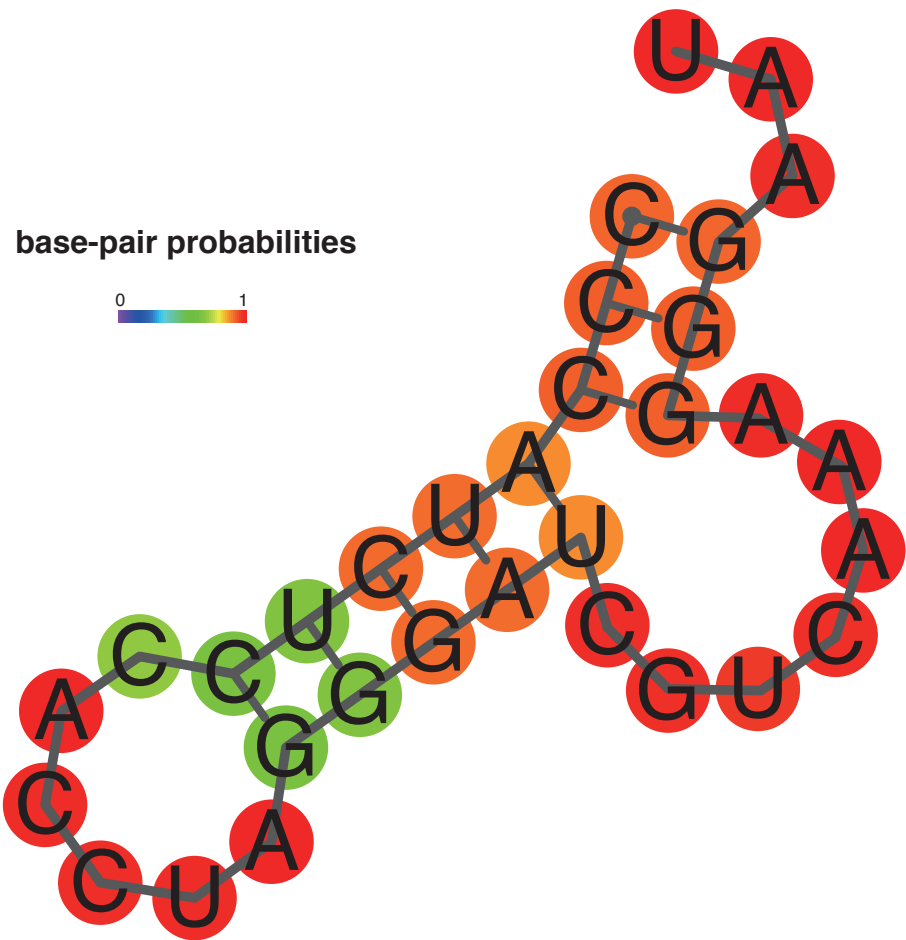

B

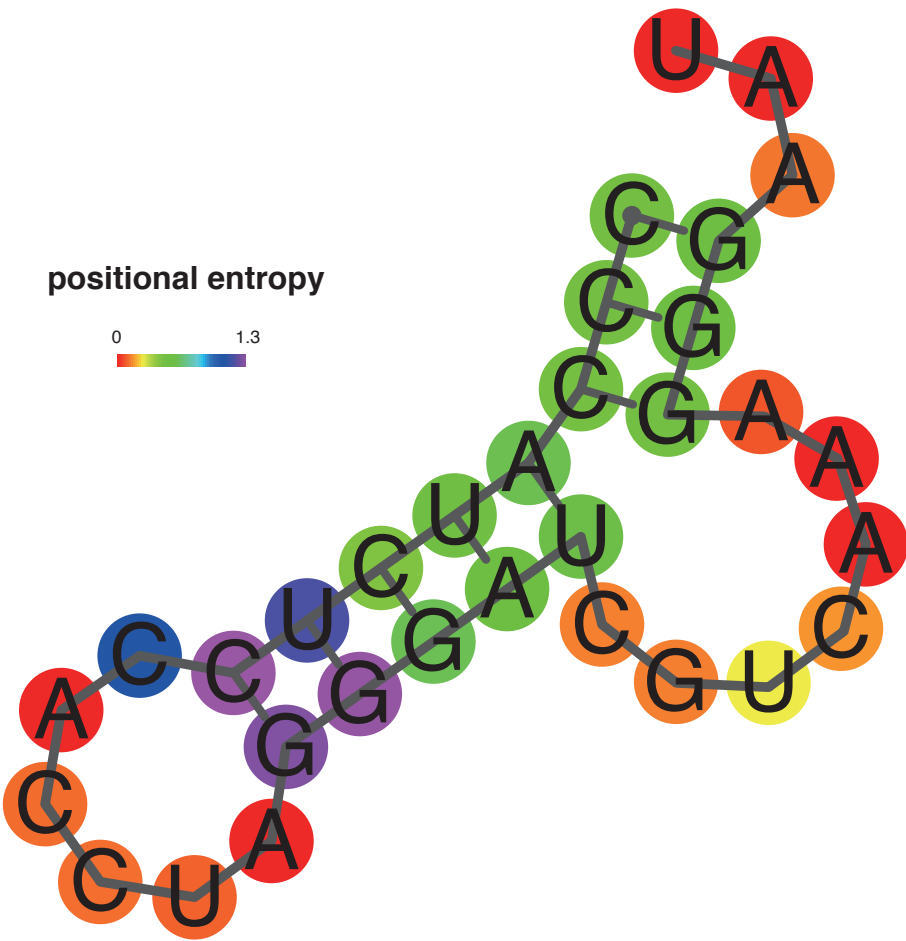

Supplement: S2 Fig — The secondary structure of the conserved 32 nucleotides RNA in hXIST RNA was predicted using the RNAfold web server (http://rna.tbi.univie.ac.at/cgi-bin/RNAWebSuite/RNAfold.cgi). The secondary structures are colored by base-pair probabilities (A) and by positional entropy (B). RNA parameters are described in Supplementary reference 2. (PDF) [file pone.0201239.s002.pdf]
